# Bridging marine microbiota ecology and IMO policy: rethinking open-loop scrubber environmental impacts

**DOI:** 10.1093/sumbio/qvaf035

**Published:** 2026-01-09

**Authors:** Savvas Genitsaris, Natassa Stefanidou, Evangelia Michaloudi, Leonidas Ntziachristos, Maria Moustaka-Gouni

**Affiliations:** Section of Ecology and Taxonomy, School of Biology, National and Kapodistrian University of Athens, Zografou Campus, 15784 Athens, Greece; Department of Botany, School of Biology, Aristotle University of Thessaloniki, 54124 Thessaloniki, Greece; Department of Zoology, School of Biology, Aristotle University of Thessaloniki, 54124 Thessaloniki, Greece; Department of Mechanical Engineering, Aristotle University of Thessaloniki, 54124 Thessaloniki, Greece; Department of Botany, School of Biology, Aristotle University of Thessaloniki, 54124 Thessaloniki, Greece

**Keywords:** biodegradation, marine microbiology, bacteria, pollutants, ecotoxicity

## Abstract

Laboratory single-species ecotoxicity tests provide early-warning indicators of hazards from shipping open-loop scrubber effluent. However, such tests, conducted under highly controlled, microbiota-free conditions, neglect contaminant interactions with diverse microbial communities (billions of cells L⁻¹) prior to encountering larger marine organisms. Extrapolating results of single-species experiments and their modeling outcomes to marine ecosystems can overestimate ecological risk and can result in policies such as banning open-loop scrubbers. In fact, scrubbers may not only result in reduced SOx emissions but may also lower atmospheric Polycyclic Aromatic Hydrocarbons (PAHs) emissions, especially in port areas, thus contributing to improved air quality. Obviously, such trade-offs between cleaner air and cleaner seas present challenges to policymakers. Mesocosm ecotoxicity experiments show bacterioplankton, phytoplankton, and metazoan plankton communities’ resilience to diluted effluent in seawater and the increase of bacterial xenobiotic biodegradation gene machinery. These experiments indicate a fast bacterial biodegradation of abundant PAHs affecting the exposure levels to the rest of marine organisms. Another key factor mitigating PAH impacts in mesocosms is diatom-dominated phytoplankton as their enlarged phycosphere is a microhabitat for PAH-degrading bacteria. Marine microbes can quickly break down pollutants and sustainable environmental risk assessment policies must weigh this natural resilience alongside the air-quality gains from scrubbers.

**Sustainability Statement**

The UN Sustainable Development Goals (SDGs) lay out the roadmap for an integrated strategy to assess the impacts of scrubber effluents on both air and water quality. A sustainable ecotoxicological approach targeting high environmental realism aligns with (i) SDG 14 (Life Below Water) by exploring microbial PAH degraders diversity and gene pool content in contamination hot spots, and assessing the PAHs attenuation in the marine environment aiming to limit their impacts and preserve bottom-up ecosystem stability, (ii) SDG 3 (Good Health and Well-being) by reducing harmful air emissions, and (iii) SDG 12 (Responsible Consumption and Production) by applying sustainable production technologies within global shipping supply chains.

## Introduction

Ships carry >80% of the volume of global trade (UNCTAD), being the most eco-friendly mode of transportation per ton of freight carried, but they remain major contributors to airborne sulfur emissions and air quality degradation (Nunes et al. 2017). To mitigate emission impacts on the environment and human health, the International Maritime Organization (IMO) set a limit on the fuel sulfur content used on board ships at 0.5% m/m, marking a significant milestone to improve air quality. Alternatively, measure ships can be equipped with Exhaust Gas Cleaning Systems (EGCS), also known as scrubbers. In open-loop scrubbers, scrubber washwater is produced after spraying fresh seawater into the EGCS and then directly released into the marine environment. The IMO (2020) sulfur cap (https://www.imo.org/en/mediacentre/hottopics/pages/sulphur-2020.aspx) has accelerated the installation of open-loop EGCS in vessels. This alternative was widely adopted with a total of 5893 vessels equipped with scrubbers by August 2024 (Miller 2024). Scrubbers have been shown to improve air quality by reducing SOx concentrations with a modelling approach (Fragkou et al. 2025) to compliant levels (Grigoriadis et al. 2024). However, as the washwater produced by open loop-scrubbers contains several contaminants, including Polycyclic Aromatic Hydrocarbons (PAHs), metals, NOx, and particles, concerns about the environmental impact of using scrubbers are raised (Gondikas et al. 2025). Specifically, the PAHs mixture can pose a threat to ecosystems and human health due to its toxicity (Barbosa et al. 2023). Thus, water quality criteria protective of marine life based on the toxicity of representative PAHs in marine test species are proposed (de Jourdan et al. 2025).

As a response to this complex environmental trade-off, the EU-funded project EMERGE (Evaluation, control, and Mitigation of the EnviRonmental impacts of shippinG Emissions) aimed to evaluate the environmental impacts of scrubbers both to air and water. Several ecotoxicological tests were performed within EMERGE by five European research labs. For these tests, the scrubber effluent during an on-board campaign of a vessel travelling from Belgium to Turkey was obtained in 12 different stations, and this effluent was similar by 80% in all samples used in the experiments (Maragkidou et al. 2024). The ecotoxicological results of single-species tests that were used for risk assessment suggested that the scrubber effluent was highly toxic at an unusually high dilution in the marine environment and could have serious implications for the populations of several species and the marine ecosystem health overall (Chen et al. 2024, Monteiro et al. 2024). Based on an increased shipping traffic scenario in the European Seas by 2050 and these single species ecotoxicity results, Aghito et al. (2025) assessed the adverse impact of scrubber use. These experimental and scenario-based model outcomes have influenced several countries and regions to implement bans, especially in ports, while OSPAR agreed to a staged ban on the release of discharge water from ships’ exhaust gas scrubbers in coastal waters of North-East Atlantic Ocean (OSPAR COMMISSION 2025). However, results of community ecotoxicology tests showed microbiota mitigation functions and no negative effects at high concentrations of the effluent contaminants due to bacterial fast degradation of the most abundant and toxic PAH contaminants (Genitsaris et al. 2025, Kourkoutmani et al. 2025).

In the EMERGE project onboard campaign, the measurements of total PAHs and alkyl-PAHs indicated that heavy fuel oil, with scrubber’s use, was outperforming ultra-low sulfur fuel at low engine loads (Grigoriadis et al. 2022). Remarkably, as part of a systematic holistic approach, a recent study based on well-to-wake life cycle assessment (LCA) and *in situ* data showed that for ocean-going bulk carriers operating in open seas under similar engine operating modes during an actual ocean voyage, heavy fuel oil used with a scrubber outperforms low-sulfur fuels (Stathatou et al. 2025). Additionally, in that work, observed values of pH, metals, NOx, and PAHs in washwater discharges were well within IMO environmental thresholds. The study underlined the need for comprehensive LCA studies to properly assess emission abatement technologies. Wu et al. (2021) suggested that the fuel switch policy could lead to greater emissions of single-ring aromatics and the potential for secondary aerosol and ozone formation, which has implications for local air quality. Implementation of a global ultra-low-sulfur oil policy soon needs to be optimized; aromatics were the most important common contributors to O_3_ and secondary organic aerosol in ship exhausts during on-board test of volatile organic compounds from nine typical cargo ships operating on low-sulfur fuels in China (Zhang et al. 2024). Neglecting pollutants such as O_3_ and secondary organic aerosols would vastly underestimate the actual influence of shipping emissions on air quality. Furthermore, the impact of the IMO sulfur reduction policy on gaseous hydrocarbon emissions, including volatile and intermediate volatility organic compounds (naphthalene and alkylated naphthalene) remains underexplored (Shukla et al. 2025). Taking into account that ship effluents are expected to be diluted by thousands to millions of times in spatiotemporal scales ranging from 2 to 25 km and in 2–60 h (Zervakis et al. 2025) while in port areas contaminants might accumulate forming hot spots of contaminants (Stathatou et al. 2025), the duality of air and water presents a policy challenge: how can regulators ensure cleaner air and clean seas, especially in port cities?

The present work provides a scientifically grounded framework for integrating conflicting ecotoxicological findings, including our own, and reflects on what this means for environmental regulation objectives on cleaner air for human health and resilient marine urban environment. Contributing to the ongoing IMO policy discussions on the role of open-loop scrubbers as an alternative transitional technology toward sustainable fuels, we argue that the value of scrubbers is being eroded. First, a brief review of the current state of evidence based on experimental findings of the EMERGE project is presented. Then, we focus on our own published results that indicate negligible impacts under community test conditions simulating high environmental realism of natural marine microbiota diversity and complexity. An example of how outcomes are not always consistent and possible explanations of why the results diverge are given. Finally, implications for scrubber effluent ecotoxicological experiments are discussed and future directions toward science-informed policy decisions based on environmentally relevant robust evidence are laid.

### The current state of evidence

Several single-species tests spanning from the bacterium *Aliivibrio fischeri* to metazoa (Picone et al. 2023) showed adverse effects at very low dilution of the scrubber effluent equivalent to the Lowest Observed Effect Concentration (LOEC) with the lowest one (0.0001%) for the sea urchin fertilization (Chen et al. 2024, Table 1). The significant effect on the fertilization of the green sea urchin (*Strongylocentrotus droebachiensis*) eggs occurred when the concentration of 16 PAHs was only 0.02 ng L^−1^ and their alkylated derivatives concentration was only 0.04 ng L^−1^ (Jalkanen et al. 2024). Similar were the results for the polychaete *Sabellaria alveolate* larval development (Monteiro et al. 2024). The significant effect on the fertilization of the purple sea urchin (*Paracentrotus lividus*) eggs and the malformation of its larvae was observed at higher concentrations (Monteiro et al. 2024). In the above-mentioned tests, according to the standard protocols, a single or few females and males were used for fertilization, the dilution water for the effluent was either filtered seawater (FSW) (0.22–0.45 μm) or synthetic seawater (SSW) and the adverse effects were observed at pg L^−1^ concentrations of the effluent’s PAHs mixture. It is worth noting that PAHs in the natural marine environment are generally present as a mixture of compounds in the low ng L^−1^ PAHs range (Martinez-Varela et al. 2022).

**Table 1.** Target organism, type of experiment, tested endpoint, and LOEC.

Reference	Target organism	Type of experiment	Endpoint	LOEC (% dilution)
Picone et al. (2023)	*Aliivibrio fischeri*	Microtox SSW	Bioluminescence	20%
Picone et al. (2023)	*Phaeodactylum tricornutum*	Single species test F2 medium	Growth rate	40%
Picone et al. (2023)	*Dunaliella tertiolecta*	Single species test F2 medium	Growth rate	20%
Picone et al. (2023)	*Acartia tonsa*	Single species test SSW	Copepod lethality Hatching F_0_ Larval development F_0_ Larval mortality F_0_ Egg production F_0_	10%–20% 20% 2% 20% 0.01%
Picone et al. (2023)	*Mytilus galloprovincialis*	Single species test SSW	Larval development	1%
Monteiro et al. (2024)	*Sabellaria alveolata*	Single species test FSW	Larval development	0.001%
Monteiro et al. (2024)	*Paracentrotus lividus*	Single species test FSW	Fertilization Larval development	1% 0.1%
Monteiro et al. (2024)	*Mytilus galloprovincialis*	Single species test FSW	Post exposure feeding inhibition	0.001%
Chen et al. (2024)	*Strongylocentrotus droebachiensis*	Single species test FSW	Fertilization	0.0001%
Genitsaris et al. (2023)	Phytoplankton and bacterioplankton Natural communities	Mesocosms NSW	Species growth rate Community growth rate Community composition Abundance	Bacterioplankton and picophytoplankton growth rate (>10%) Nano- micro-phytoplankton growth rate (10%) Bacterioplankton and phytoplankton composition shifts (>10%) Phytoplankton abundance (10%)
Genitsaris et al. (2024)	Phytoplankton and bacterioplankton Natural communities	Mesocosms NSW	Species growth rate Communitygrowth rate Community composition Abundance Genes linked to PAHs degradation	Bacterioplankton and picophytoplankton growth rate (>10%) Nano- micro-phytoplankton growth (10%) Bacterioplankton and phytoplankton composition shifts (>10%) Phytoplankton abundance (10%) PAH degradation complete modules (10%)
Genitsaris et al. (2025)	Phytoplankton and bacterioplankton Natural communities	Mesocosms NSW	Species growth rate Community growth rate Community composition Abundance Genes linked to PAHs degradation	Bacterioplankton and picophytoplanktongrowth rate (>5%) Nano- micro-phytoplankton growth rate (5%) Bacterioplankton and phytoplankton composition shifts (>5%) Phytoplankton abundance (5%) PAH degradation complete modules (10%)
Kourkoutmani et al. (2025)	Metazooplankton Natural community	Mesocosms NSW	Growth rate Community composition Abundance Early-life stages of metazoa	Metazoan total growth (10%) Adult growth (>10%) Early developmental stages growth (10%)

*NSW* Natural Seawater.

As an example of how ecotoxicological experiments’ outcomes are not always consistent even when using scrubber effluent of the same vessel (with chemical composition with similarity >80%), below we briefly outline our own published data (Genitsaris et al. 2023,^2024^,^2025^, Kourkoutmani et al. 2025). Mesocosm experiments examining the effects of four different dilutions (1, 2, 5, and 10% v/v) of the scrubber effluent on the marine environment were performed in triplicate treatments to study interconnected natural communities of phytoplankton and bacterioplankton by applying an ecosystem-based approach and using microscopy, metabarcoding and metagenomics analysis for taxonomic and functional endpoints. Bacterioplankton, phytoplankton, and zooplankton populations exhibited growth in controls validating CRED criteria (Moermond et al. 2016). The experimental exposure time of 6 days (longer than the standard 3 days) was the maximum to avoid any mesocosm artifact from wall-growth effect. A high number of organisms per replicate was used for all controls and test concentrations (at least thousands of individuals were identified and counted per sample). Hundreds of Operational Taxonomic Units (OTUs) of bacterioplankton interacting with several OTUs of phytoplankton were exposed to the same controlled conditions of scrubber effluent input. Metazoan plankton in presence of natural bacteria and phytoplankton were also exposed to the same scrubber effluent. The species tested were fully relevant for the compartment under evaluation (water column). PAHs concentrations, microscopy population abundance and metabarcoding read abundance data of triplicates were checked with the Levene’s test for variances among the replicates of the same treatment with no statistical differences among replicates of the same treatments reported. The Mann–Whitney nonparametric test was applied to evaluate whether the median of the microbial communities’ abundances as well as the dominant species abundance median were statistically different between pairs of samples (control vs treatments).

**Figure 1.**
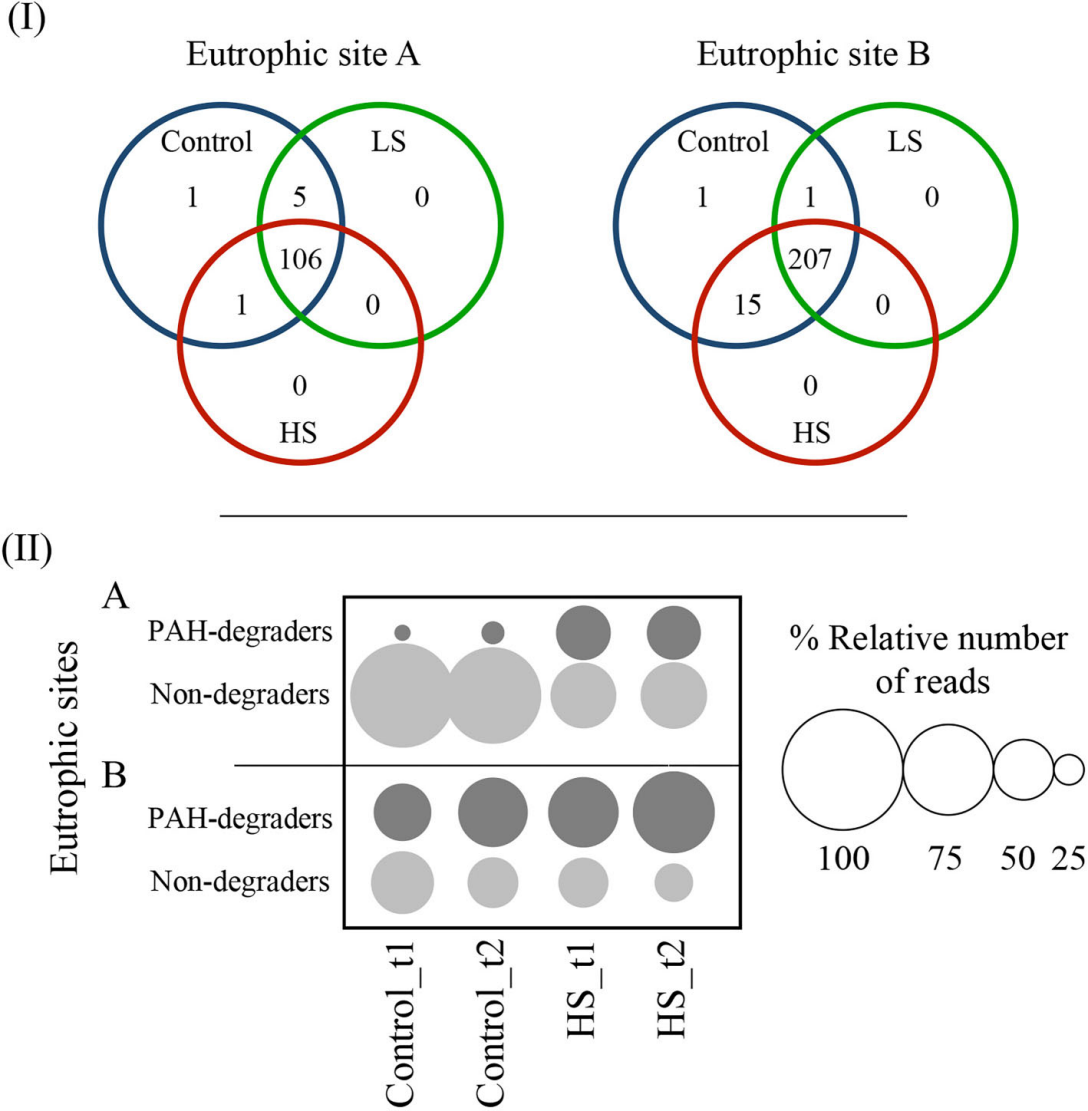
Venn diagram indicating the number of unique and shared bacterial OTUs between the different experimental treatments (I); Shifts in the relative abundance of PAH-degrader OTUs and nondegraders between treatments (II). LS denotes low scrubber dilution treatments (1%) and HS high scrubber dilution (10% in eutrophic site A & 5% in eutrophic site B).

The results indicated negligible impacts under test conditions with LOEC at 5%–10% v/v dilution for nano- and micro-phytoplankton growth endpoint, 10% v/v for metazoan early life stages growth and >10% v/v for adults’ growth, and >10% v/v for picophytoplankton and bacterioplankton growth (Table 1). Slight or no differences in the taxonomic composition of bacterial OTUs were inferred by 16S rRNA gene sequencing for dominant OTUs (relative abundance ≥0.01% in the dataset). The Venn diagram showing the number of unique and shared OTUs between the different scrubber water treatments, indicates the high shared biodiversity (106 OTUs–93.8%) among the control and the 1% (LS) and 10% (HS) scrubber water treatments in the tested eutrophic site A (Thessaloniki Bay, Northern Greece) (Fig. 1). High number of shared OTUs were also recorded among the control and the 1% (LS) and 5% (HS) scrubber water treatments in the tested eutrophic site B (Saronikos Gulf, Athens, Greece). In particular, 207 out of 224 OTUs (92.4%) were common in the three treatments (Fig. 1). The minor differences in the taxonomic composition of bacterial OTUs in bacterioplankton communities after exposure to the contaminants cocktail suggest communities’ resistance with shift toward increased PAHs degraders abundances (Fig. 1) and not due to the replacement of sensitive species by more tolerant ones (Blanck 2002).

**Figure 2.**
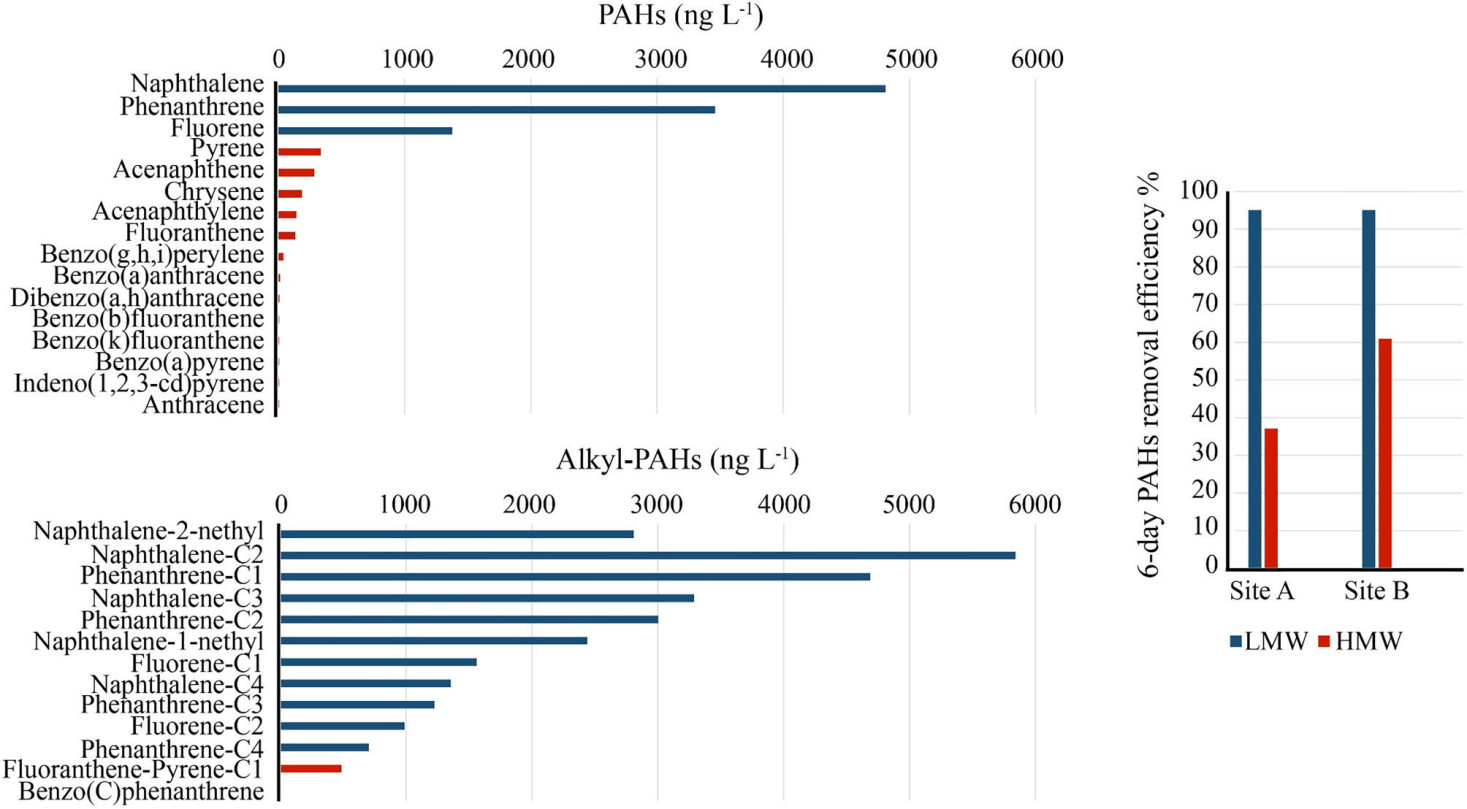
PAHs and alkyl-PAHs in the scrubber effluent used in community ecotoxicology experiments. Insert indicates 6 days % removal of LMW (in blue) and HMW PAHs (in red) in urban site experiments A and B.

Furthermore, the bacterial community shifts to effluent exposure in both tested sites coincided with rapid declines in PAH and alkyl-PAH concentrations during diatom blooms. Following the protocols for PAHs measurements described by García-Gómez et al. (2024), the 6th day removal efficiency of most PAHs and alkyl-PAHs of the effluent assigned to Low Molecular Weight (LMW) PAHs and alkyl-PAHs was very high, exceeding 90% (Fig. 2). Considerable was the removal efficiency of High Molecular Weight (HMW) PAHs and alkyl-PAHs. In site B mesocosm test communities, the high (61%) removal of HMW PAHs coincided with the diatom *Skeletonema costatum* bloom. This diatom species producing extracellular polymeric substances increased the size and stickiness of phycosphere, a biotope of PAHs-degrading bacteria and thus could stimulate mineralization rates of total PAHs by passively concentrating these contaminants in the marine water (Gutierrez et al. 2014).

The experiments indicated a fast bacterial ultimate biodegradation of most abundant PAHs affecting the exposure levels to the rest of marine organisms from phytoplankton to multicellular biota, reducing the potential adverse effects on metazoan plankton (Kourkoutmani et al. 2025). The observed high functional-response diversity of PAHs degrading bacteria reflected the high potential for acute mitigation responses of scrubber effluent experimental phytoplankton and metazoan plankton communities. This can be critical for the preservation of community resistance and bottom-up ecosystem stability (Genitsaris et al. 2023). Dombrowski et al. (2016) showed that the repertoire of PAHs use varied among different bacterial taxa and the synergistic capabilities of the microbial community exceeded those of individual taxa indicating that effective PAHs degradation processes require diverse and complementary capacities of natural microbiota.

### Why do the results diverge between approaches?

Methodological considerations, such as the design of single-species ecotoxicology vs. mesocosm community ecotoxicology tests are key factors for results divergence. Mesocosms are a valuable tool to bridge the scale gap between single-species laboratory experiments and field studies (Moustaka-Gouni et al. 2016). Unlike single-species flask experiments, our mesocosms encompassed the natural diversity of bacterioplankton, phytoplankton, and metazoans. Consequently, experimental conditions were selected to reflect the best-adapted genotypes from diverse, initial interconnected communities rather than from a single species population. While mesocosm responses cannot replicate long-term, large-scale community shifts to pollutants, the 6-day duration effectively captured rapid responses, multidomain interactions, bloom dynamics, and shifts in species dominance. The experiment exceeded standard algal exposure durations of 72 h (TGD27; EC, 2018) and 94 h (OECD, 2006).

Natural bacterioplankton and phytoplankton exposed to shipping scrubber effluent responded within 24 h. Genitsaris et al. (2023, 2024, 2025) indicated that the source bacterial community structure was a key factor to biodegradation initialization when bacterial OTUs and genes involved in PAHs and alkyl-PAHs degradation were conspicuous. Another key factor to buffering the impacts of effluent PAHs in our mesocosms was the diatom-dominating phytoplankton surface-water partitioning and its carbon biomass. Mineralization of PAHs can be significantly enhanced in the presence of diatom cell surfaces. Even though marine phytoplankton communities, in general, do not degrade PAHs themselves, they are considered a global reservoir of PAHs degraders (MARPAH 2011) acting as hosts and creating favorable conditions for bacterial degradation through nutrient release, surface provision, and oxygenation, leading to the natural attenuation of PAHs in marine systems. PAHs microbial metabolic capacity is a widespread trait in the global upper ocean. Martinez-Varella et al. (2022) indicated microbial control over the fate of the background PAHs concentrations in the surface ocean, with PAHs degradation genes being ubiquitous in oceanic metagenomes (González-Gaya et al. 2019). Therefore, we highlight that the key point of why results diverge between mesocosm vs single-species approaches examining the impacts of scrubber effluents is the absence of natural microbiota that adsorb, degrade, or transform PAH contaminants in the natural environments, making single-species test organisms more vulnerable than in realistic ocean conditions.

**Figure 3.**
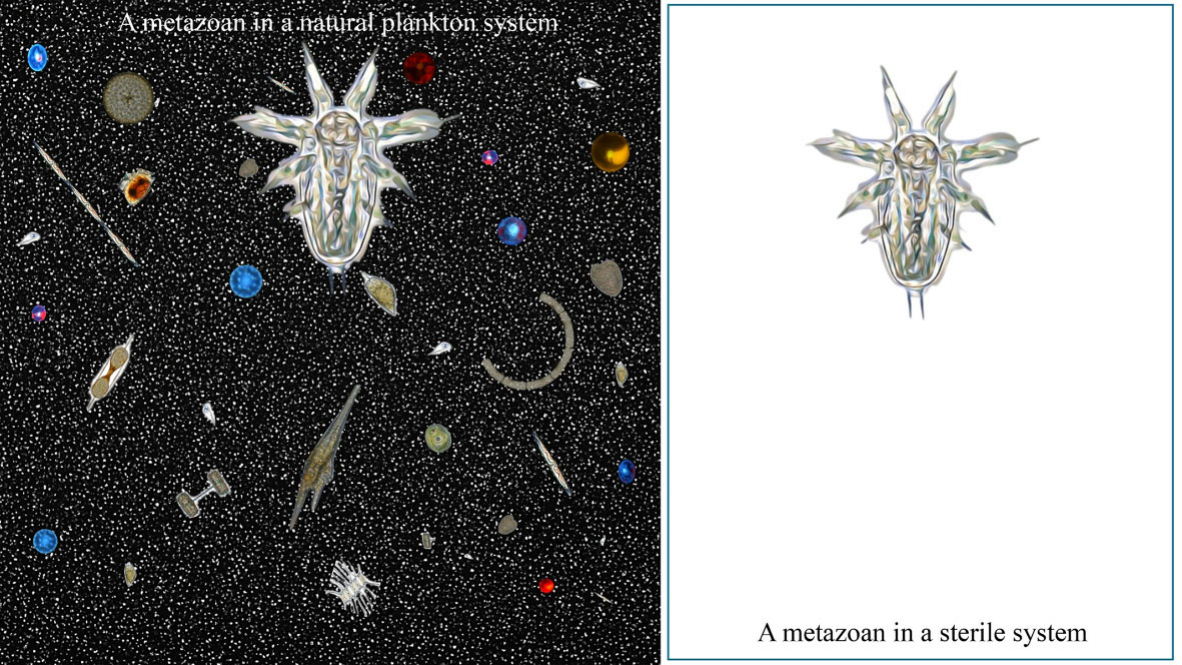
A visual explanation to question ecological relevance of standard tests without microbial context. Representation of the relative abundance of marine bacteria, algae, and early-life stage metazoan in the euphotic zone. Bacteria: the white dots on the black background, Phytoplankton: diverse micrographs.

Fig. 3 provides a visual explanation for the divergence of results and highlights why the ecological relevance of single-species tests is questionable when assessing the impacts of scrubber effluent contaminants in natural systems, where abundant microbiota mediates the initial phase of contaminant fate. Ignoring marine microbiota (bacteria and protists) overlooks the first millions of live interacting encounters that a contaminant faces in the marine environment.

### Implications and future perspectives

Microorganisms can be regarded as miniature bags of enzymes, finely tuned to sense their environment and to degrade and recycle the products of human activity (Pearce 2024). In the marine water column, their abundances can reach billions of cells L^−1^ having direct and immediate contact with dissolved and particulate contaminants, indicating their potential to solve some of the world’s greatest pollution challenges through their bioremediation machinery (Miglani et al. 2022). Assessing the impacts of open-loop scrubber effluent commands not overlooking the interactive effects of bacterioplankton and phytoplankton communities. By integrating responses of diverse microbial species, community ecotoxicology provides realistic insights into effects on metazoa and contaminant mixture degradation. This has led to a viewpoint shift of ecotoxicological approaches toward community-level responses (Clements and Rohr 2009). Marine ecosystems with interconnected communities of bacterioplankton, phytoplankton, and planktonic metazoa, with high functional diversity, are more likely to recover after effluent disturbances. In contrast, polluted areas such as ports, where functional diversity is lower (Genitsaris et al. 2023) and contaminants may accumulate (Stathatou et al. 2025), are more vulnerable to the combined effects of open-loop discharges. Nevertheless, network analysis of bacterial communities exposed to 1% v/v effluent—several orders of magnitude higher than the expected 0.0001% dilution in the vicinity of the discharge in the marine environment (Zervakis et al. 2025)—showed connectivity patterns like those of controls at the port area (Genitsaris et al. 2023). The bacterial communities' network collapsed only at 10% v/v treatments, a dilution that is unrealistic. Phytoplankton diversity, abundance, and dominance remained unchanged in port areas at 1% v/v treatments, indicating that bacterioplankton buffering have possibly enhanced phytoplankton resistance to stressors. Additionally, large micro-phytoplankton dominants in polluted areas, with bigger phycosphere radii, likely provide larger biotope for associated PAH-degrading bacteria and PAHs detoxication (Gutierrez et al. 2014).

To achieve sustainability policy targets, future ecotoxicological experiments on scrubber effluents must shift from current single-species tests toward integrated evidence from field observations and community-level ecotoxicology. As up to date experiments that advise scrubber bans are testing only a couple of effluent samples on few metazoan individuals in natural microbiota-free conditions, they naturally fail to assess the role of microbial-mediated contaminant degradation, and the ecosystem resilience mechanisms remain overlooked. Incorporating microbial community interactions into global initiatives, such as the UN SDGs (Ramos et al. 2024), and risk assessments can improve predictions of effluent impacts, guide safe discharge limits, and strengthen protection of vulnerable coastal ecosystems. To ensure regulations are scientifically sound, evidence-based and environmentally effective, several effluent samples from a representative set of ships (containers, bulk carriers, passenger ferries) operating on different types of fuel oil and fitted with open scrubbers need to be fueled into natural communities from locations of variable trophic status and pollution stressors. Several ecological endpoints must be assessed, including microorganism community structure and functioning endpoints, gene pool content and enzyme activity, while also not forgetting the atmospheric impacts of any technologies considered. Importantly, the benefits of scrubbers in reducing air emissions and protecting human health should not be overlooked, especially in port cities. Policymakers should also evaluate the full “well-to-wake” life cycle impacts of shipping technologies (Stathatou et al. 2025) to ensure regulations are balanced across ecological, health, and socio-economic dimensions.

## Data Availability

No new data were generated or analysed in support of this article.

## References

[bib1] Aghito M, Majamäki E, Hänninen R et al. Projected changes of the emission and transport of organic pollutants and metals from shipping in European seas 2018–2050. Mar Pollut Bull. 2025;211:117351. 10.1016/j.marpolbul.2024.117351. 39674034

[bib2] Barbosa JF, Rocha BA, Souza MCO et al. Polycyclic aromatic hydrocarbons (PAHs): updated aspects of their determination, kinetics in the human body, and toxicity. J Toxicol Environ Health, Part B. 2023;26:28–65. 10.1080/10937404.2022.2164390. 36617662

[bib3] Blanck H. A critical review of procedures and approaches used for assessing pollution-induced community tolerance (PICT) in biotic communities. Human Ecolog Risk Assessment: Int J. 2002;8:1003–34. 10.1080/1080-700291905792.

[bib4] Chen CY, Magnusson K, Pfeiffer R et al. Exhaust gas cleaning system effluents from ships impair fertilization and larval development in the green sea urchin *Strongylocentrotus droebachiensis* at very low concentrations. Available at SSRN: 5015537. 2024. 10.1021/acs.est.5c02483 PMC12947621 41655137

[bib5] Clements WH, Rohr JR. Community responses to contaminants: using basic ecological principles to predict ecotoxicological effects. Environ Toxicol Chem. 2009;28:1789–800. 10.1897/09-140.1. 19358627

[bib6] de Jourdan BP, Daniel DL, Gardinali PR et al. Toxicity of six representative polycyclic aromatic compounds in five marine test species. Sci Total Environ. 2025;986:179574. 10.1016/j.scitotenv.2025.179574. 40450776

[bib7] Dombrowski N, Donaho JA, Gutierrez T et al. Reconstructing metabolic pathways of hydrocarbon-degrading bacteria from the Deepwater Horizon oil spill. Nat Microbiol. 2016;1:16057. 10.1038/nmicrobiol.2016.57. 27572965

[bib40_429_093626] European Commission. 2018. Directorate-General for Health and Food Safety, Technical Guidance for Deriving Environmental Quality Standards –, European Commission. https://data.europa.eu/doi/10.2875/018826

[bib8] Fragkou E, Tsegas G, Alyuz U et al. Assessing the efficiency of different mitigation strategies to reduce shipping related air pollution levels and exposure in the Mediterranean coastal region—an ensemble modelling approach. Atmos Environ. 2025;360:121347. 10.1016/j.atmosenv.2025.121347.

[bib9] García-Gómez E, Isna S, Gros M et al. Rapid and sensitive method for the simultaneous determination of PAHs and alkyl-PAHs in scrubber water using HS-SPME-GC–MS/MS. MethodsX. 2024;12:102589. 38322135 10.1016/j.mex.2024.102589 PMC10844971

[bib10] Genitsaris S, Kourkoutmani P, Stefanidou N et al. Effects from maritime scrubber effluent on phytoplankton and bacterioplankton communities of a coastal area. Ecol Inform. 2023;77:102154. 10.1016/j.ecoinf.2023.102154.

[bib11] Genitsaris S, Stefanidou N, Hatzinikolaou D et al. Marine microbiota responses to shipping scrubber effluent assessed at community structure and function endpoints. Environ Toxicol Chem. 2024;43:1012–29. 10.1002/etc.5834. 38415986

[bib12] Genitsaris S, Stefanidou N, Kourkoutmani P et al. Do coastal bacterioplankton communities hold the molecular key to the rapid biodegradation of Polycyclic Aromatic Hydrocarbons (PAHs) from shipping scrubber effluent?. Environ Res. 2025;277:121563. 10.1016/j.envres.2025.121563. 40203979

[bib13] Gondikas A, Mattsson K, Hassellöv M. A new form of hazardous microparticulate contamination to the marine environment from ships using heavy fuel oil with exhaust gas scrubbers–Characterization and implications for fate, transport and ecotoxicity. Sci Total Environ. 2025;959:178263. 10.1016/j.scitotenv.2024.178263. 39756303

[bib14] González-Gaya B, Martínez-Varela A, Vila-Costa M et al. Biodegradation as an important sink of aromatic hydrocar-bons in the oceans. Nat Geosci. 2019;12:119–25. 10.1038/s41561-018-0285-3.

[bib15] Grigoriadis A, Kousias N, Raptopoulos A et al. EMERGE Deliverable 3.1, “Compilation and analysis of experimental data from onboard campaigns, including emission and activity data and profiles. 2022.

[bib16] Grigoriadis A, Kousias N, Raptopoulos-Chatzistefanou A et al. Particulate and gaseous emissions from a large two-stroke slow-speed marine engine equipped with open-loop scrubber under real sailing conditions. Atmosphere. 2024;15:845. 10.3390/atmos15070845.

[bib17] Gutierrez T, Rhodes G, Mishamandani S et al. Polycyclic aromatic hydrocarbon degradation of phytoplankton-associated *Arenibacter* spp. and description of *Arenibacter algicola* sp. nov., an aromatic hydrocarbon-degrading bacterium. Appl Environ Microb. 2014;80:618–28. 10.1128/AEM.03104-13. PMC3911095 24212584

[bib18] IMO. Cutting sulphur oxide emissions. 2020. https://www.imo.org/en/mediacentre/hottopics/pages/sulphur-2020.aspx (15 December 2025, date last accessed).

[bib19] Jalkanen JP, Fridell E, Kukkonen J et al. Environmental Impacts of Exhaust Gas Cleaning Systems in the Baltic Sea, North Sea, and the Mediterranean Sea Area. Helsinki, Finland: Finnish Meteorological Institute, 2024. 10.35614/isbn.9789523361898.

[bib20] Kourkoutmani P, Genitsaris S, Demertzioglou M et al. Effects from maritime scrubber effluent on coastal metazooplankton. Mar Biol. 2025;172:2. 10.1007/s00227-024-04562-8.

[bib21] Maragkidou A, Jalkanen JP, Hänninen R et al. EMERGE Deliverable 9.4. “Final report to the EC summing up achievements of project, including financial report.” 2024.

[bib22] MARPAH Marine Micro-Algae as Global Reservoir of Polycyclic Aromatic Hydrocarbon Degraders. 2011. https://cordis.europa.eu/article/id/88619-marine-algae-host-spillbusting-bacteria?utm_source. (19 October 2025, date last accessed).

[bib23] Martinez-Varela A, Casas G, Berrojalbiz N et al. Polycyclic aromatic hydrocarbon degradation in the sea-surface microlayer at coastal Antarctica. Front Microbiol. 2022;13:907265. 10.3389/fmicb.2022.907265. 35910648 PMC9329070

[bib24] Miglani R, Parveen N, Kumar A et al. Degradation of xenobiotic pollutants: an environmentally sustainable approach. Metabolites. 2022;12:818. 10.3390/metabo12090818. 36144222 PMC9505297

[bib25] Miller G. Shipowners still adding more scrubbers, via newbuildings not retrofits. Lloyd’s List. 2024. https://www.lloydslist.com/LL1150318/Shipowners-still-adding-more-scrubbers-via-newbuildings-not-retrofits.

[bib26] Moermond CT, Kase R, Korkaric M et al. CRED: criteria for reporting and evaluating ecotoxicity data. Environ Toxicol Chem. 2015;35:1297–309. 10.1002/etc.3259. 26399705

[bib27] Monteiro A, Rodrigues V, Picado A et al. Holistic evaluation of the environmental impacts of shipping in the sensitive region of Ria de Aveiro. Sci Total Environ. 2024;946:174314. 10.1016/j.scitotenv.2024.174314. 38944305

[bib28] Moustaka-Gouni M, Kormas KA, Scotti M et al. Warming and acidification effects on planktonic heterotrophic pico-and nanoflagellates in a mesocosm experiment. Protist. 2016;167:389–410. 10.1016/j.protis.2016.06.004. 27472657

[bib29] Nunes R, Alvim-Ferraz M, Martins F et al. Assessment of shipping emissions on four ports of Portugal. Environ Pollut. 2017;231:1370–9. 10.1016/j.envpol.2017.08.112. 28917818

[bib39_363_093426] OECD. 2006. OECD Guidelines for the Testing of Chemicals. 201: Freshwater alga and cyanobacteria, growth inhibition test. Organization for Economic Cooperation and Development, Paris, p. 25

[bib30] OSPAR COMMISSION. 2025. The OSPAR Acquis: Decisions, recommendations and other agreements that constitute the accumulated body of OSPAR measures and actions. https://www.ospar.org/news/ministers-meet-in-vigo-to-take-concrete-measures-to-enhance-protection-of-the-north-east-atlantic-ocean.

[bib31] Pearce DA. Announcing sustainable microbiology: how microbes make a sustainable world. Sustain Microbiol. 2024;1:qvae001. 10.1093/sumbio/qvae001. PMC13375312 42576878

[bib32] Picone M, Russo M, Distefano GG et al. Impacts of exhaust gas cleaning systems (EGCS) discharge waters on planktonic biological indicators. Mar Pollut Bull. 2023;190:114846. 10.1016/j.marpolbul.2023.114846. 36965268 PMC10152311

[bib33] Ramos LJ, de Lorenzo V, López P. Meeting report: microbes as safeguards of the environment. Sustain Microbiol. 2024;1:qvae013. 10.1093/sumbio/qvae013. PMC13409297 42576915

[bib34] Shukla D, Czech H, Kokkola T et al. Emission speciation of volatile and intermediate volatility organic compounds from a marine engine: effect of engine load, fuel type and photochemical aging. Environ Sci: Atmosph. 2025;5:973–86.

[bib35] Stathatou PM, Petrunia I, Barenthin T et al. Marine scrubbers vs low-sulfur fuels: a comprehensive well-to-wake life cycle assessment supported by measurements aboard an ocean-going vessel. Environ Sci Technol. 2025;59:7066–80. 10.1021/acs.est.4c10006. 40183449 PMC12004910

[bib37] Wu J, Posen ID, MacLean HL. Trade-offs between vehicle fuel economy and performance: evidence from heterogeneous firms in China. Energy Policy. 2021;156:112445. 10.1016/j.enpol.2021.112445.

[bib38] Zervakis V, Kolovoyiannis V, Calgaro L et al. A novel method to assess the dilution of complex mixtures in the marine environment: application to marine scrubber water effluents. Mar Pollut Bull. 2025;216:117956. 10.1016/j.marpolbul.2025.117956. 40239280

[bib39] Zhang F, Xiao B, Liu Z et al. Real-world emission characteristics of VOCs from typical cargo ships and their potential contributions to secondary organic aerosol and O3 under low-sulfur fuel policies. Atmos Chem Phys. 2024;24:8999–9017. 10.5194/acp-24-8999-2024.

